# Volatile Fingerprinting and Interpretable Machine Learning for Quality Differentiation of Astragali Radix from Different Cultivation Patterns

**DOI:** 10.3390/foods15152624

**Published:** 2026-07-27

**Authors:** Shulin Yu, Ziyue Song, Yunqi Sun, Wanying Li, Jiayi Dong, Huiqin Zou, Yonghong Yan

**Affiliations:** School of Chinese Materia Medica, Beijing University of Chinese Medicine, Beijing 102488, China; yushulin0115@163.com (S.Y.); 20240935226@bucm.edu.cn (Z.S.);

**Keywords:** Astragali Radix, volatile fingerprint, HS-SPME-GC–MS, HS-GC–IMS, cultivation pattern, quality differentiation, interpretable machine learning, SHAP

## Abstract

Volatile fingerprints provide useful information for characterizing Astragali Radix (AR), a food–medicine homologous plant material, but differences among wild, wild-simulated, and cultivated samples remain unclear. In this study, headspace solid-phase microextraction coupled with gas chromatography–mass spectrometry (HS-SPME-GC–MS) and headspace gas chromatography–ion mobility spectrometry (HS-GC–IMS) were integrated with multivariate analysis and interpretable machine learning to characterize volatile profiles and identify candidate discriminatory compounds in 117 AR samples from different cultivation patterns. HS-SPME-GC–MS tentatively identified 29, 34, and 45 volatile compounds in wild, wild-simulated, and cultivated samples, respectively. Esters were the predominant class in all groups, although the relative abundance of esters and the overall chemical-class composition varied among cultivation patterns. HS-GC–IMS tentatively identified 57, 50, and 55 compounds, respectively, comprising mainly low-molecular-weight aldehydes, alcohols, and ketones and thereby providing complementary volatile fingerprint information. Partial least squares discriminant analysis (PLS-DA) showed that the volatile fingerprints captured cultivation-pattern-associated differences, with the HS-GC–IMS model showing clearer group separation. Random forest, support vector machine, and CatBoost models were further constructed using the HS-SPME-GC–MS profiling results. By integrating variable importance in projection (VIP) and SHapley Additive exPlanations (SHAP) values, γ-hexalactone, methyl eugenol, methyl (9Z,11E)-octadeca-9,11-dienoate, eugenol, and ethyl linoleate were selected as candidate discriminatory compounds. Based on the HS-GC–IMS results, 1-octen-3-one, pentyl acetate, (Z)-2-penten-1-ol, 2-heptanone, and the monomeric signal of 2-ethyl-6-methylpyrazine were also identified as candidate discriminatory compounds. These compounds may be related to fatty acid-derived metabolism, aromatic secondary metabolism, and terpenoid-related processes. The integration of two complementary volatile-analysis platforms with VIP- and SHAP-based interpretation provided broader coverage of volatile features and improved the interpretability of candidate-compound screening. These findings provide an interpretable analytical workflow and candidate discriminatory compounds that may support future rapid screening, cultivation-pattern authentication, and volatile-profile-based differentiation of AR, pending independent external validation.

## 1. Introduction

Astragali Radix (AR), the dried root of *Astragalus membranaceus* (Fisch.) Bge. var. *mongholicus* (Bge.) Hsiao or *Astragalus membranaceus* (Fisch.) Bge., is a widely used Chinese herbal medicine with a long history of medicinal use. In addition to its traditional medicinal value, AR is regarded in China as a typical medicine–food homologous material. It was classified as a legal dietary supplement under the U.S. Dietary Supplement Health and Education Act (DSHEA) in 1994 [1], and was included in the Chinese catalogue of medicine–food homology in 2019. In recent years, AR and its extracts have been increasingly used in food, cosmetic, and animal husbandry industries, for example as functional food ingredients [2,3], cosmetic ingredients [4], and feed additives [5,6]. These applications are generally associated with its diverse bioactive constituents and health-promoting properties. Therefore, AR is an important plant material for both traditional quality evaluation and modern food-related applications.

The quality of AR is closely related to its cultivation pattern and growing environment. Similar to Panax ginseng, AR is mainly obtained from three production modes: wild, wild-simulated, and cultivated [7]. Previous studies have shown that wild and wild-simulated AR have similar patterns of chemical constituent accumulation and pharmacological activity, and they are generally considered to have quality characteristics closer to each other than to cultivated material [8,9]. The growth cycle also differs markedly among these types, usually exceeding 10 years for wild plants, more than 5 years for wild-simulated plants, and approximately 2 years for cultivated plants, which may lead to differences in secondary metabolite accumulation [10]. In recent years, considerable progress has been made in comparing AR from different cultivation patterns, mainly in relation to amino acid metabolism, saponin biosynthesis, and flavonoid metabolism [11,12]. However, the volatile characteristics of AR have received less attention, although odor is an important sensory attribute in the traditional appraisal of Chinese medicinal materials. AR occurs in wild, wild-simulated, and cultivated forms, which differ in growth environment, metabolic activity, and chemical composition [13]. These differences may result in distinct volatile fingerprints. Clarifying cultivation-pattern-associated volatile variation is therefore important for supporting the quality differentiation and cultivation-pattern authentication of AR [13]. These differences may result in distinct volatile fingerprints. Clarifying cultivation pattern-related volatile variation is therefore important for improving the quality differentiation and cultivation-pattern authentication of AR.

Headspace solid-phase microextraction coupled with gas chromatography–mass spectrometry (HS-SPME-GC–MS) is widely used for the qualitative and semi-quantitative analysis of volatile compounds, while headspace gas chromatography–ion mobility spectrometry (HS-GC–IMS) offers rapid analysis, high sensitivity, and minimal sample preparation, especially for low-molecular-weight volatiles [14]. Owing to their different analytical characteristics, these two techniques can provide complementary information for characterizing complex volatile profiles, as reported in recent volatile-profile studies [15,16,17]. However, volatile datasets generated by these platforms are usually high-dimensional and contain many correlated variables, making it necessary to use appropriate data-analysis methods. Recent studies have shown that machine learning can be combined with volatile fingerprints to support pattern recognition, feature selection, and candidate-compound screening [18,19]. Moreover, Shapley additive explanations (SHAP)-based interpretable machine learning has been increasingly applied in metabolomics and volatile-profile analysis to quantify the contributions of individual variables, improve model transparency, and support the identification of candidate discriminatory features [20,21]. Despite these advances, systematic comparisons of the complementary volatile fingerprints of AR from different cultivation patterns remain limited, and the contributions of individual volatile compounds to sample differentiation have not been sufficiently interpreted. Therefore, an integrated workflow combining complementary volatile profiling with interpretable machine learning is needed to characterize cultivation-pattern-associated differences and identify the principal volatile contributors.

Accordingly, this study aimed to (1) characterize the complementary volatile fingerprints of wild, wild-simulated, and cultivated AR using HS-SPME-GC–MS and HS-GC–IMS; (2) evaluate cultivation-pattern-associated differences using multivariate analysis and machine learning; and (3) identify and interpret candidate discriminatory compounds by integrating VIP and SHAP analyses.

## 2. Materials and Methods

### 2.1. Preparation of Samples

A total of 117 Astragali Radix samples were collected from September to October in 2023 and 2024 and naturally air-dried after harvesting. All samples were authenticated as Astragalus membranaceus (Fisch.) Bge. var. mongholicus (Bge.) Hsiao by Professor Yong-Hong Yan of Beijing University of Chinese Medicine according to the Chinese Pharmacopoeia (2020 edition). According to growth mode, cultivation records, and plant age, the samples were classified as wild (*n* = 24), wild-simulated (*n* = 32), and cultivated (*n* = 61). Wild samples grew naturally without artificial cultivation, wild-simulated samples were established under near-natural conditions with limited human intervention, and cultivated samples were grown under conventional field management. Wild and wild-simulated samples were more than 5 years old, whereas cultivated samples were 1–2 years old. The wild and wild-simulated samples originated from Shanxi Province, while the cultivated samples were collected from Shanxi (*n* = 16), Gansu (*n* = 17), and Inner Mongolia (*n* = 28). Before volatile analysis, all 117 samples were pulverized and passed through a No. 4 sieve. The unequal group sizes reflected the actual availability of Astragali Radix samples from different cultivation patterns in the commercial and production settings.

### 2.2. HS-SPME-GC-MS Analysis

#### 2.2.1. Extraction of Volatile Organic Compounds (VOCs)

The HS-SPME procedure was adapted from Chen et al. [22], with modifications to the fiber coating, equilibration temperature and time, extraction time, and desorption conditions. Each AR sample was pulverized and passed through a No. 4 pharmacopoeial sieve. An accurately weighed portion of 1.0 g was transferred into a 20 mL headspace vial and immediately sealed with a PTFE/silicone septum and an aluminum crimp cap. The samples were extracted using headspace solid-phase microextraction (HS-SPME). Briefly, the sealed vials were equilibrated at 80 °C for 30 min with stirring at 250 rpm. An 80/10 μm divinylbenzene/carboxen/polydimethylsiloxane (DVB/CAR/PDMS) fiber was then exposed to the headspace for 50 min at an insertion depth of 40 mm under continuous agitation. After extraction, the fiber was immediately inserted into the GC inlet for thermal desorption for 10 s.

#### 2.2.2. GC–MS Determination

Volatile compounds were analyzed using an Agilent 8890 gas chromatograph coupled with an Agilent 7000E triple-quadrupole mass spectrometer (Agilent Technologies, Santa Clara, CA, USA) operated in full-scan mode. Chromatographic separation was performed using two HP-5MS UI capillary columns connected in series, each measuring 30 m × 0.32 mm i.d. with a film thickness of 0.25 μm. The oven temperature was initially maintained at 40 °C for 2 min, increased to 160 °C at 4 °C/min and held for 2 min, and subsequently increased to 250 °C at 10 °C/min and held for 2 min. The inlet temperature was maintained at 250 °C, and samples were introduced in splitless injection mode. High-purity helium (99.999%) was used as the carrier gas at a constant flow rate of 1.0 mL/min. The mass spectrometer was operated in electron ionization mode at 70 eV, with the ion source and quadrupole temperatures maintained at 230 and 150 °C, respectively. Full-scan mass spectra were acquired over an m/z range of 20–600.

### 2.3. HS-GC-IMS Analysis

A 1.00 g portion of each sample was placed in a 20 mL headspace vial and analyzed using a FlavourSpec^®^ gas chromatography–ion mobility spectrometry system (G.A.S. Gesellschaft für analytische Sensorsysteme mbH, Dortmund, Germany) equipped with a CTC-PAL 3 static headspace autosampler (CTC Analytics AG, Zwingen, Switzerland). The sealed vials were incubated at 80 °C for 15 min with agitation at 500 rpm. Chromatographic separation was performed using an MXT-WAX capillary column (30 m × 0.53 mm i.d., 1.0 μm film thickness). The column temperature was maintained at 60 °C, and ultra-high-purity nitrogen (≥99.999%) was used as the carrier gas. The carrier-gas flow rate was initially maintained at 2.0 mL/min for 2 min, increased linearly to 10.0 mL/min over 8 min, subsequently increased to 150.0 mL/min over 10 min, and maintained at 150.0 mL/min for 20 min, resulting in a total run time of 40 min. The injector temperature was set at 80 °C. The ion mobility spectrometer was equipped with a tritium (^3H) ionization source and a 98 mm drift tube maintained at 45 °C with an electric field strength of 500 V/cm. Ultra-high-purity nitrogen (≥99.999%) was used as the drift gas at a flow rate of 150 mL/min, and the measurements were performed in positive ion mode.

### 2.4. Data Processing

#### 2.4.1. HS-SPME–GC-MS Data Processing

Each sample was independently weighed and analyzed three times as independent analytical replicates. Analytical repeatability was evaluated using the relative standard deviation (RSD) values of representative peaks, with the results and peak-selection criteria provided in Table S4. Volatile compounds were tentatively identified by comparison with the NIST 23 mass spectral library using a minimum match score of 70%. Because authentic standards and experimentally determined retention indices were not used, all compound assignments were considered tentative. Relative abundance was calculated by peak-area normalization. No internal standard was used; therefore, the results represent relative semi-quantitative data rather than absolute concentrations. The resulting data matrix was used for subsequent statistical analysis and machine learning.

#### 2.4.2. HS-GC-IMS Data Processing

Each sample was independently weighed and analyzed three times as independent analytical replicates. A retention-index calibration curve was established using a mixed standard solution of six ketones. Compound identification was performed in VOCal software by matching the calculated retention indices and drift times against the NIST 2020 gas chromatographic retention index database and the ion mobility spectrometry drift time database. Analytical repeatability was evaluated using the RSD values of representative peaks, with the results and peak-selection criteria provided in Table S3.

### 2.5. Statistical and Machine Learning Analysis

The HS-SPME-GC-MS and HS-GC-IMS results were expressed as “mean ± standard deviation.” Differences among the three cultivation groups were evaluated using the Kruskal–Wallis nonparametric test in SPSS 26.0 (SPSS Inc., Chicago, IL, USA). A value of *p* < 0.05 was considered statistically significant.

#### 2.5.1. Partial Least Squares Discriminant Analysis (PLS-DA)

Before partial least squares discriminant analysis (PLS-DA), the datasets were transformed using log10(x + 1) and subjected to Pareto scaling. PLS-DA was performed in RStudio (version 2025.09.2+418). A maximum of 10 latent variables was evaluated. The optimal number was selected according to the highest Q^2^ obtained by leave-one-out cross-validation. Model performance was evaluated using R^2^X, R^2^Y, and Q^2^. Model validity was further assessed using 200 permutation tests.

#### 2.5.2. Machine Learning Analysis

Machine learning analysis was performed in Python (version 3.13.1) using the dataset derived from the 117 samples. Data splitting was performed at the sample level. The three analytical replicates from each sample were kept within the same training, validation, or test subset to prevent data leakage. The data were divided into training, validation, and test subsets at a ratio of 60:20:20 using stratified random splitting. The random seed was set to 42. Class weighting was applied to reduce the influence of unequal group sizes. No resampling method, including the Synthetic Minority Over-sampling Technique (SMOTE), was used. Hyperparameters were optimized using five-fold grid-search cross-validation (GridSearchCV) with accuracy as the scoring metric. For the random forest model, the tested numbers of trees were 100 and 200, the maximum depths were 10, 20, and unrestricted, and the minimum split sizes were 2 and 5. The optimal settings were 200 trees, a maximum depth of 10, and a minimum split size of 5. For the radial basis function (RBF) support vector machine, C values of 0.1, 1, and 10 and gamma values of “scale” and “auto” were evaluated. The optimal settings were C = 10 and gamma = “scale”. For CatBoost, the tested numbers of iterations were 100 and 200, the depths were 4, 6, and 8, the learning rates were 0.05 and 0.10, and the L2 regularization values were 1, 3, and 5. The optimal settings were 100 iterations, a depth of 4, a learning rate of 0.10, and an L2 regularization value of 1.

SHAP were used to interpret the models. TreeExplainer was applied to the random forest and CatBoost models, whereas KernelExplainer was used for the support vector machine. For multiclass classification, the absolute SHAP values were averaged across observations within each class and then across the three classes. Model generalization and potential overfitting were evaluated by comparing performance among the training, validation, and test subsets.

## 3. Results and Discussion

### 3.1. Volatile Profiles of AR with Different Cultivation Patterns

#### 3.1.1. HS-SPME-GC-MS Profiling

**Figure 1:**
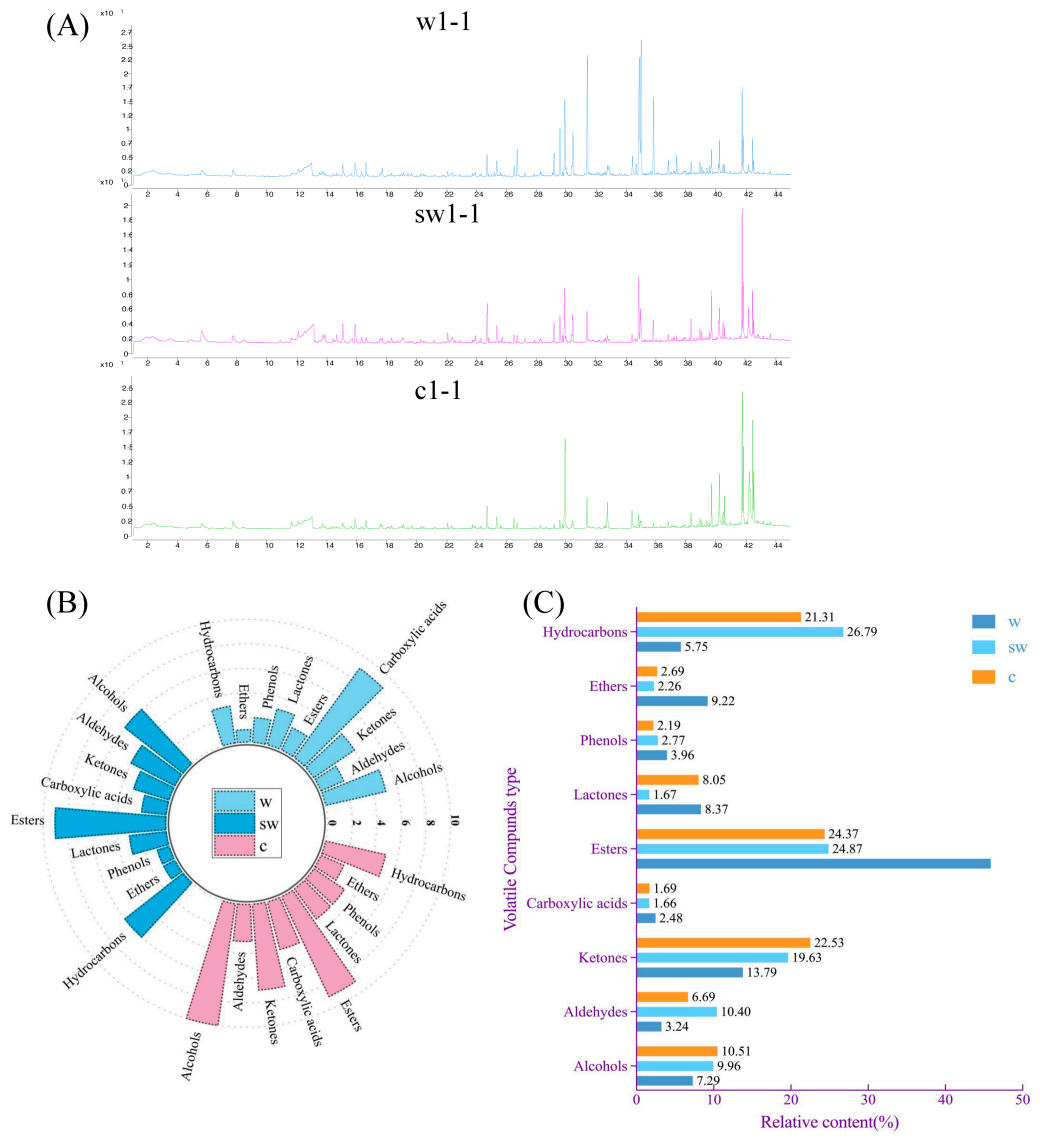
HS-SPME-GC-MS analysis of volatile compounds in AR samples with three different cultivation patterns. (**A**) Volatile fingerprints of wild (w), wild-simulated (sw), and cultivated (c) AR samples; (**B**) composition and chemical-class distribution of volatile compounds in AR samples with different cultivation patterns; (**C**) relative contents of different classes of volatile compounds in AR samples with different cultivation patterns.

The HS-SPME-GC–MS volatile fingerprints of AR samples from the three cultivation patterns are shown in Figure 1A. Overall, the chromatographic profiles of wild and wild-simulated samples were relatively similar, whereas cultivated samples showed more evident differences from the other two groups. As shown in Figure 1B, 29, 34, and 45 volatile compounds were tentatively identified in wild, wild-simulated, and cultivated samples, respectively. These compounds included esters, alcohols, aldehydes, ketones, acids, lactones, phenols, ethers, and hydrocarbons. More volatile compounds were detected in the cultivated group. This may partly reflect its larger sample size, which was designed to represent the predominance of cultivated Astragali Radix in the current market. Differences in plant age, geographical origin, cultivation management, environmental exposure, and stress-related metabolism may also have contributed to this observation, although their individual effects could not be distinguished in the present study.

The relative distribution of different chemical classes further showed cultivation pattern-related differences (Figure 1C). Esters were the predominant class in all three groups, but their relative abundance varied markedly. Wild samples had the highest proportion of esters (45.88%), which was substantially higher than that in wild-simulated and cultivated samples, both of which were approximately 24%. Ketones and lactones were relatively more abundant in wild samples, hydrocarbons were more prominent in wild-simulated samples, and ketones and hydrocarbons represented important proportions of the volatile profile of cultivated samples. According to the compound identification results in Table S1, the major volatile compounds also differed among the three groups. Wild samples were mainly characterized by methyl (9Z,11E)-octadeca-9,11-dienoate, methyl linolenate, methyl eugenol, ar-turmerone, and γ-nonalactone, while wild-simulated samples showed higher relative levels of β-sesquiphellandrene, ar-turmerone, hexanal, and (E, E)-3,5-octadien-2-one. Cultivated samples were mainly characterized by β-sesquiphellandrene, 5-acetyl-2-methoxyphenyl acetate, methyl (9Z,11E)-octadeca-9,11-dienoate, hexanal, and γ-nonalactone.

#### 3.1.2. HS-GC-IMS Profiling

**Figure 2:**
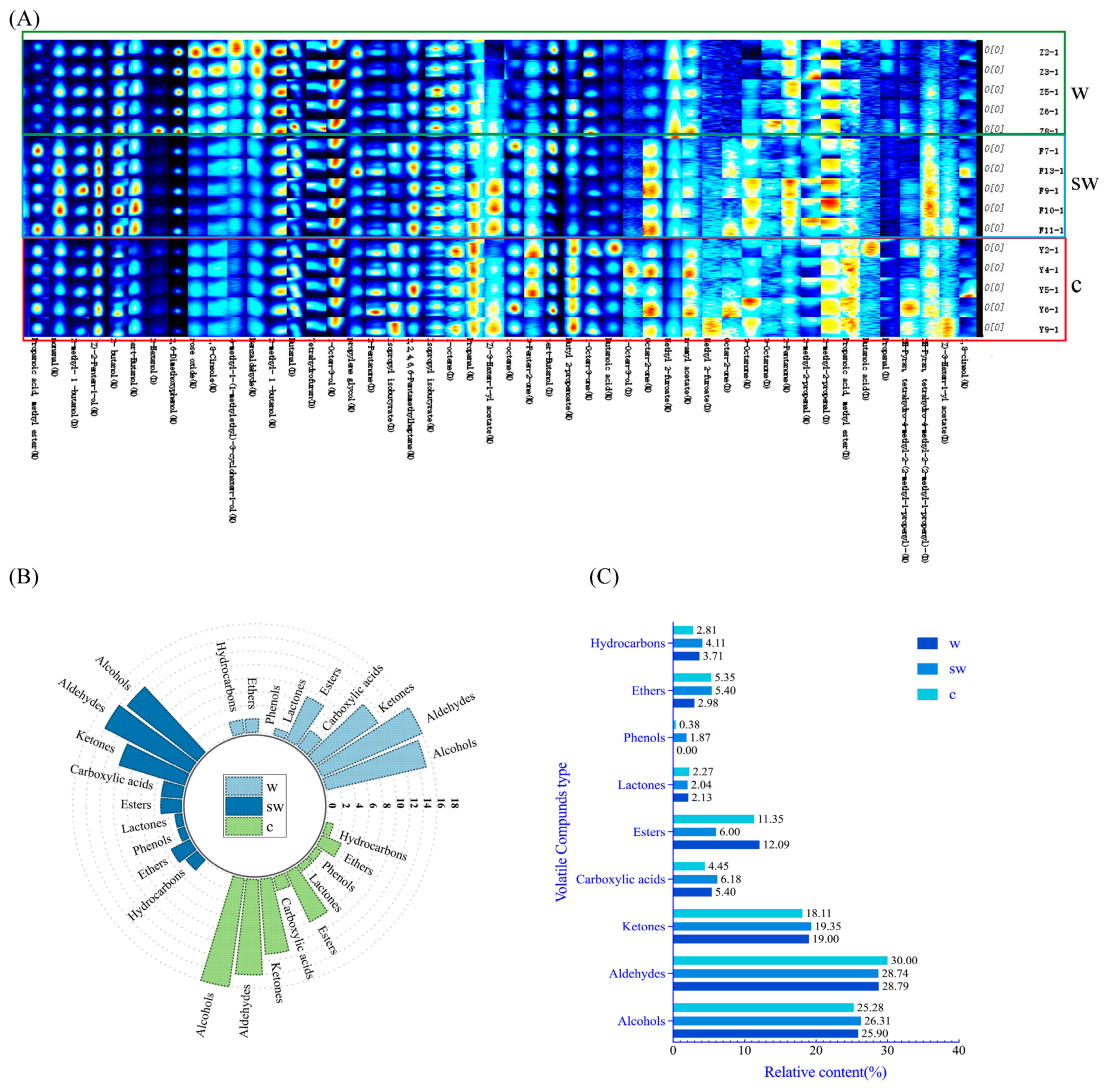
HS-GC-IMS analysis of volatile compounds in AR samples with three different cultivation patterns. (**A**) Volatile fingerprints of wild (w), wild-simulated (sw), and cultivated (c) AR samples; (**B**) composition and chemical-class distribution of volatile compounds in AR samples with different cultivation patterns; (**C**) relative contents of different classes of volatile compounds in AR samples with different cultivation patterns. In panel (A), the color gradient from blue to red represents increasing signal intensity. The colored outlines deline-ate the cultivated (c), wild-simulated (sw), and wild (w) groups.

The HS-GC–IMS volatile fingerprints of AR samples from the three cultivation patterns are shown in Figure 2A. The representative fingerprints indicated differences in the relative abundance of volatile compounds among wild, wild-simulated, and cultivated samples. Compared with HS-SPME-GC–MS, HS-GC–IMS detected a larger number of volatile compounds, with 57, 50, and 55 compounds identified in wild, wild-simulated, and cultivated samples, respectively. These volatiles were mainly low-molecular-weight compounds, particularly aldehydes, alcohols, and ketones, suggesting that the three groups shared a broadly similar basic volatile profile.

As shown in Figure 2B,C, aldehydes, alcohols, and ketones were the predominant chemical classes in all three groups. Aldehydes accounted for 28.79%, 28.74%, and 30.00% of the total volatiles in wild, wild-simulated, and cultivated samples, respectively; alcohols accounted for 25.90%, 26.31%, and 25.28%; and ketones accounted for 19.00%, 19.35%, and 18.11%, respectively. In contrast, minor chemical classes showed clearer differences among the three groups. Esters were more abundant in wild samples (12.09%) and cultivated samples (11.35%) than in wild-simulated samples (6.00%), which was generally consistent with the HS-SPME-GC–MS results. Carboxylic acids and ethers were relatively more abundant in wild-simulated samples, lactones varied only slightly among the three groups, phenols were detected at low levels and only in wild-simulated and cultivated samples, and hydrocarbons remained at relatively low levels in all groups.

According to the compound identification results in Table S2, several volatiles, including 1-butanol, 2-methyl-1-propanol, 2-heptanone, heptanal, 2-heptenal, nonanal, γ-butyrolactone, and methyl hexanoate, were detected in all three groups, suggesting that these compounds may represent common volatile constituents of AR. Despite this shared background, the dominant volatiles differed among cultivation patterns. Wild samples were mainly characterized by terpinen-4-ol, 2-heptanone, 2-heptenal, 3-octanone, and nonanal; wild-simulated samples showed relatively higher levels of 2-methylbutanoic acid, benzaldehyde, eucalyptol, and 2,6-dimethoxyphenol; and cultivated samples were mainly characterized by 2-methyl-2-pentenal, 2-methyl-1-butanol, 2,4-heptadienal, 3-methyl-1-butanol, hexanal, and ethyl hexanoate. These results indicate that HS-GC–IMS effectively captured differences in low-molecular-weight volatiles among AR samples and provided complementary information to HS-SPME-GC–MS for subsequent multivariate analysis. The median RSD values of the representative HS-SPME-GC–MS peaks ranged from 4.46% to 8.56%, while those of the representative HS-GC–IMS peaks ranged from 5.18% to 8.57%, indicating relatively limited analytical variability for the selected signals. Therefore, the large within-group standard deviations observed for some compounds likely reflected sample heterogeneity, although analytical variability could not be completely excluded.

### 3.2. Multivariate Analysis of Volatile Profiles

#### 3.2.1. PLS-DA Separation of Wild, Wild-Simulated, and Cultivated Samples

**Figure 3:**
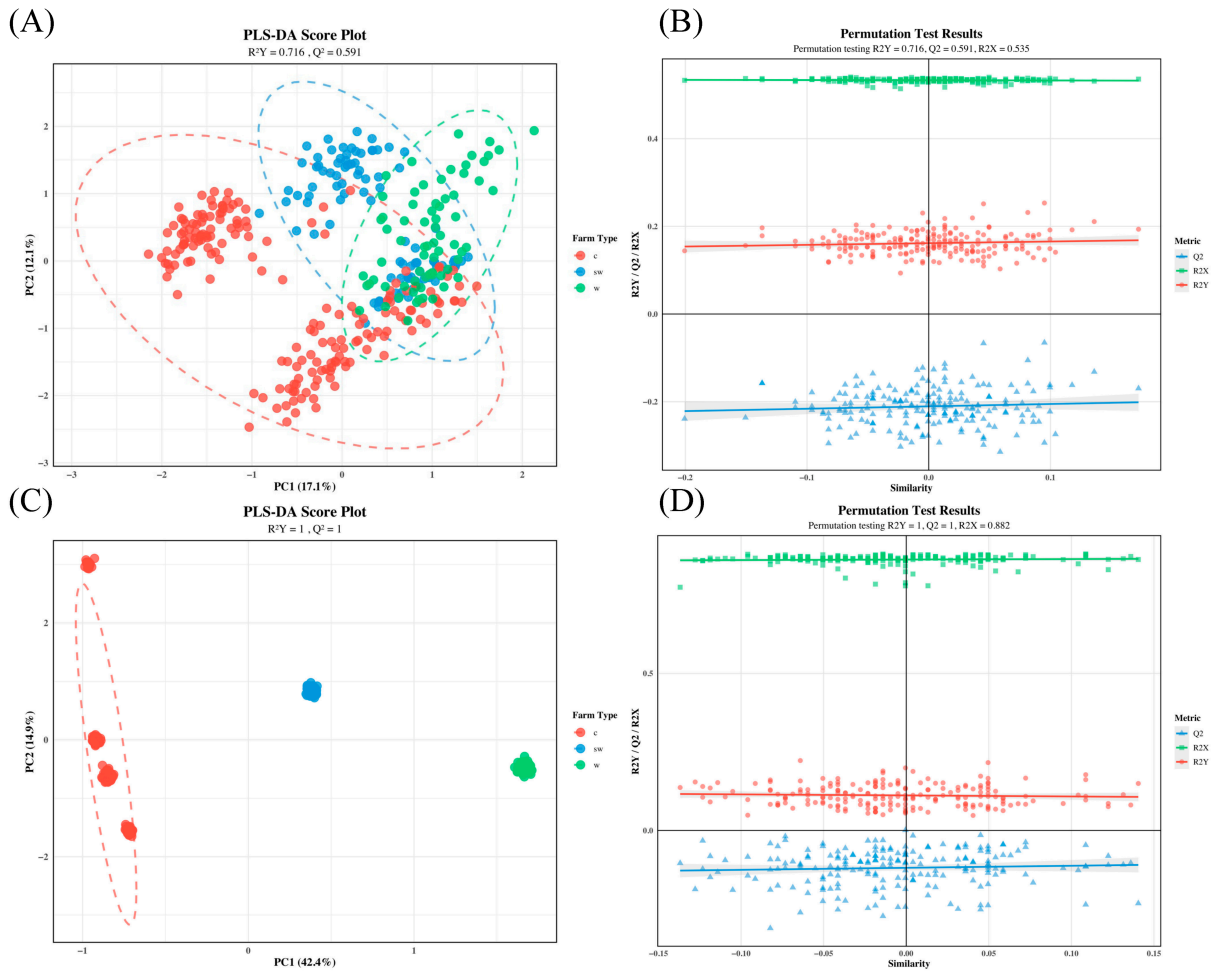
PLS-DA analysis of volatile compounds in AR samples with different cultivation patterns. (**A**,**B**) PLS-DA score plot and permutation-test results based on the HS-SPME-GC–MS dataset; (**C**,**D**) PLS-DA score plot and permutation-test results based on the HS-GC–IMS dataset. Dashed ellipses in panels (**A**,**C**) represent the 95% confidence regions of the three cultivation groups.

Based on the peak area data obtained from HS-SPME-GC–MS and HS-GC–IMS for 117 AR samples, PLS-DA models were established after log transformation and Pareto scaling. The PLS-DA score plots and corresponding permutation-test results are shown in Figure 3. For the HS-SPME-GC–MS dataset, the model yielded R^2^Y = 0.7160, Q^2^ = 0.5910, and R^2^X = 0.5354. The R^2^X value indicated that the model explained 45.60% of the total variation in the dataset, leaving a substantial proportion of the chemical variation unexplained. The score plot showed a general separation trend among wild, wild-simulated, and cultivated samples, although partial overlap remained among groups. These results suggest that the HS-SPME-GC–MS volatile profile captured cultivation-pattern-associated differences, but the moderate explained variance and group overlap require cautious interpretation.

For the HS-GC–IMS dataset, the PLS-DA model showed higher model parameters, with R^2^Y = 0.9997, Q^2^ = 0.9997, and R^2^X = 0.8824. The model was constructed using 351 analytical measurements obtained from 117 samples, with three independently prepared analytical replicates for each sample. The score plot showed clearer clustering within groups and more distinct separation among the three cultivation patterns, indicating that the small-molecule volatile fingerprints captured by HS-GC–IMS provided stable information for sample differentiation. The 200-permutation test showed that the original R^2^Y and Q^2^ values were higher than those of all randomly permuted models (empirical *p* < 0.005), supporting that the observed discrimination was unlikely to result from random class assignment. However, the exceptionally high R^2^Y and Q^2^ values should still be interpreted cautiously because the model included analytical replicates from the same samples and was not externally validated. Overall, HS-GC–IMS showed a stronger ability to reflect volatile differences among AR samples, whereas HS-SPME-GC–MS provided complementary information on more complex volatile constituents. The complementary volatile fingerprints obtained from HS-SPME-GC–MS and HS-GC–IMS therefore provided a useful basis for subsequent marker screening and quality differentiation analysis.

#### 3.2.2. VIP-Based Screening of Differential Volatile Compounds

**Figure 4:**
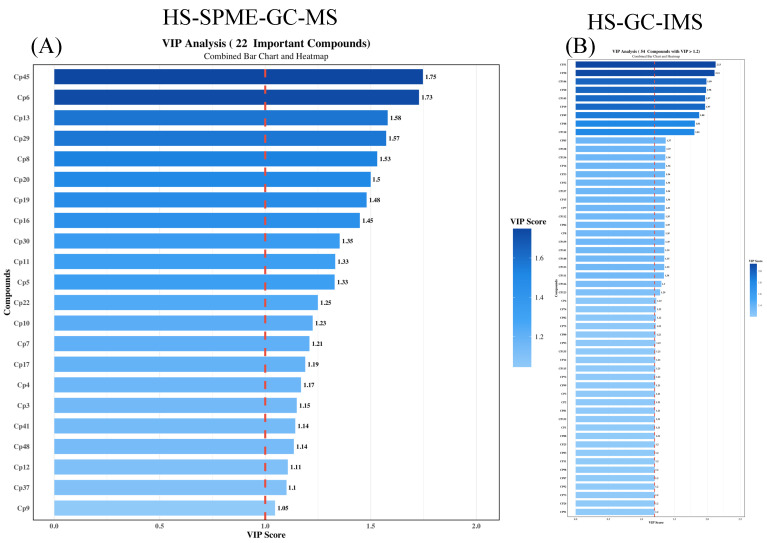
VIP analysis of the PLS-DA models for volatile compounds in AR samples with different cultivation patterns. (**A**) VIP plot based on the HS-SPME-GC–MS dataset; (**B**) VIP plot based on the HS-GC–IMS dataset. The red dashed lines indicate the VIP thresholds of 1.0 in panel (**A**) and 1.2 in panel (**B**).

Candidate differential variables from the two datasets were preliminarily screened and ranked according to the VIP values derived from the PLS-DA models, using VIP > 1 as the threshold. For the HS-SPME-GC–MS dataset, 24 candidate discriminatory volatile compounds were screened (Figure 4A). These 24 candidate discriminatory volatile compounds are summarized in Table 1. The top five compounds ranked by VIP were eugenol, 5-acetyl-2-methoxyphenyl acetate, tetradecane, benzyl alcohol, and eugenol acetate, indicating that aromatic compounds, phenolic derivatives, and hydrocarbons contributed substantially to the volatile differences among the three cultivation patterns. For the HS-GC–IMS dataset, both monomeric and dimeric signals were retained during variable screening because the same volatile compound may appear in different ion forms. This treatment helped preserve the original fingerprint information and capture the overall variation in the IMS dataset. A total of 80 variables with VIP > 1 were initially obtained and then merged into 40 candidate discriminatory compounds after combining monomeric and dimeric signals assigned to the same compound. The resulting 40 candidate discriminatory compounds are summarized in Table 2. Among these GC–IMS-derived compounds, 1-octen-3-one (CP30/CP31) showed VIP values greater than 2 for both signal forms, suggesting a strong contribution to sample differentiation. In addition, pentyl acetate, (Z)-2-penten-1-ol, 2-heptanone, and 2-ethyl-6-methylpyrazine (M) all showed VIP values above 1.5, suggesting that they were important candidate compounds for further marker screening and aroma characterization. Several high-VIP variables were not detected in one or more cultivation groups. Their VIP values may therefore partly reflect presence–absence effects rather than continuous quantitative variation. Accordingly, these variables were interpreted as candidate discriminatory features rather than quantitative biomarkers.

### 3.3. Interpretable Machine Learning Analysis of HS-SPME-GC–MS Data

Because of the complexity of volatile peak signals and the potential nonlinearity of cultivation pattern-related differences, the PLS-DA model based on the HS-SPME-GC–MS dataset showed partial overlap among the three groups. This suggested that linear latent-variable analysis alone was not sufficient to fully capture the volatile variation among AR samples. Therefore, supervised machine learning models were further introduced to improve pattern recognition and support the screening of key volatile markers. Combined with SHAP-based interpretation, these models were used to evaluate the contribution of individual volatile compounds to sample differentiation, thereby providing interpretable evidence for the quality evaluation of AR.

#### 3.3.1. Performance of Machine Learning Models

Because of the complexity of volatile peak signals and the potential nonlinearity of intergroup differences, the PLS-DA model based on the HS-SPME-GC–MS dataset showed partial overlap among the three cultivation groups. This suggested that linear latent-variable analysis alone was not sufficient to fully capture the volatile variation among AR samples. This suggested that linear latent-variable analysis alone was not sufficient to fully capture the volatile variation among AR samples. Therefore, supervised machine learning models were further introduced to characterize nonlinear differentiation patterns and support the screening of candidate discriminatory compounds. Combined with SHAP-based interpretation, these models were used to evaluate the contribution of individual volatile compounds to sample differences, thereby providing a basis for interpretable candidate-compound screening.

**Figure 5:**
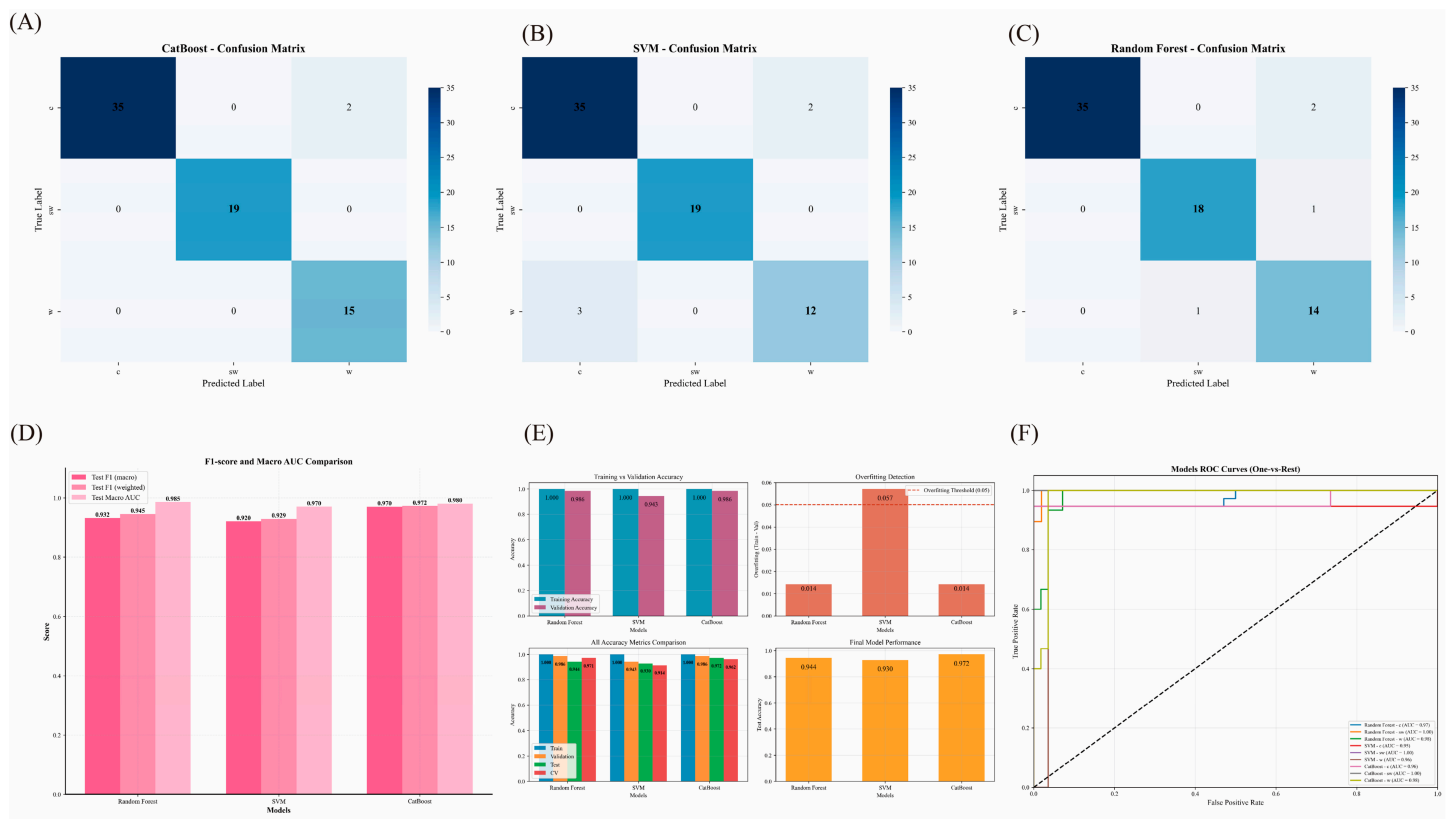
Performance evaluation of machine-learning models based on the HS-SPME-GC–MS dataset. (**A**–**C**) Confusion matrices of the CatBoost, SVM, and random forest models on the test set; (**D**) comparison of macro-F1, weighted-F1, and macro-AUC values on the test set; (**E**) comparison of training and validation accuracy, training–validation gaps, accuracy across the training, validation, and test sets, and final test accuracy; (**F**) one-vs-rest ROC curves of the three models on the test set.

Based on the volatile compound data from 117 samples, three machine learning models, Random Forest, SVM, and CatBoost, were constructed. Considering the multiclass nature of the dataset and the imbalance in sample size among groups, model performance was evaluated using confusion matrices, accuracy, macro-F1, weighted-F1, and macro-AUC. Model parameters were optimized using GridSearchCV, and potential overfitting was assessed by comparing model performance across the training, validation, and test subsets. As shown in Figure 5A–C, all three models showed consistent sample differentiation, with only a small number of incorrectly assigned samples. Incorrect assignments mainly occurred between the cultivated and wild groups, whereas the wild-simulated group was more consistently distinguished across the three models.

CatBoost achieved the highest accuracy and F1-scores, with a test accuracy of 0.972, macro-F1 of 0.970, weighted-F1 of 0.972, and macro-AUC of 0.980 (Figure 5D). Random Forest also performed well, with an accuracy of 0.944, macro-F1 of 0.932, weighted-F1 of 0.945, and macro-AUC of 0.985, whereas SVM showed relatively lower performance, with an accuracy of 0.930, macro-F1 of 0.920, weighted-F1 of 0.929, and macro-AUC of 0.970. All three models achieved a training accuracy of 1.000. However, SVM showed a larger training–validation gap of 0.057, indicating a greater degree of performance decline between the training and validation subsets. Random Forest and CatBoost showed smaller gaps of 0.014, suggesting more consistent performance across these subsets (Figure 5E). One-vs-rest ROC analysis further showed good separability among the three groups, with an AUC of 1.00 for the wild-simulated class across all models and AUC values of 0.95–0.98 for the cultivated and wild classes (Figure 5F). Overall, CatBoost showed the best balance among accuracy, F1-score, and performance consistency and was therefore selected for subsequent SHAP-based interpretation. Nevertheless, because the analytical replicates were entered separately and no independent external dataset was used, the reported model performance should be interpreted cautiously.

#### 3.3.2. SHAP-Based Interpretation of Volatile Compound Contributions

**Figure 6:**
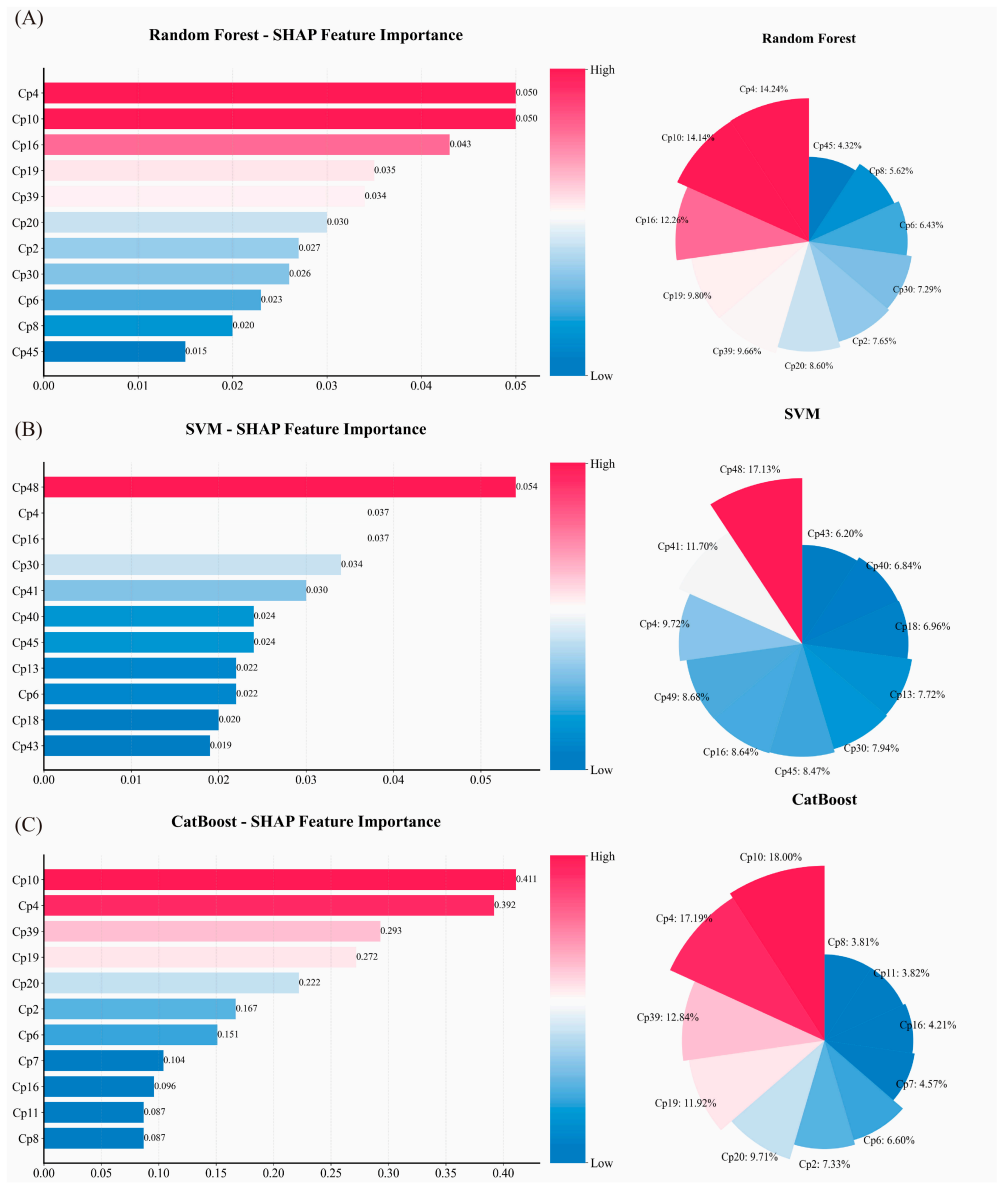
Key volatile features identified by different machine learning models and their SHAP contribution analysis. (**A**–**C**) SHAP importance analysis of the top 10 feature variables identified by the Random Forest, SVM, and CatBoost models, respectively. The bar plots on the left show the mean absolute SHAP values of each feature, which quantify their contributions to the model-based sample differentiation results, whereas the pie charts on the right show the relative contribution proportions of the corresponding features among the top 10 important variables in each model.

SHAP analysis was used to evaluate the contribution of each volatile feature to the machine learning models and to identify the features most strongly associated with cultivation pattern-related differences. As shown in Figure 6, the three models identified a group of high-contribution volatile features, and several core features were generally consistent across models. Taking CatBoost, which showed the best overall balance of accuracy and stability, as an example, methyl eugenol (Cp10) and γ-hexalactone (Cp4) made the greatest contributions, followed by eugenol (Cp39), ethyl linoleate (Cp19), and benzyl alcohol (Cp20). These features played important roles in explaining the volatile differences among wild, wild-simulated, and cultivated AR samples. The SHAP ranking obtained from Random Forest was largely consistent with that of CatBoost, further supporting the stability of these key features. Although the SVM ranking differed to some extent from those of the tree-based models, γ-hexalactone (Cp4) and methyl (9Z,11E)-octadeca-9,11-dienoate (Cp16) consistently showed high contribution levels across all three models. Overall, the SHAP results indicated that the volatile differences among AR samples were mainly associated with a limited number of high-contribution features, among which methyl eugenol, γ-hexalactone, and methyl (9Z,11E)-octadeca-9,11-dienoate may serve as representative candidate markers for quality differentiation and cultivation-pattern authentication.

#### 3.3.3. Integrated VIP-SHAP Screening of Candidate Volatile Markers

**Figure 7:**
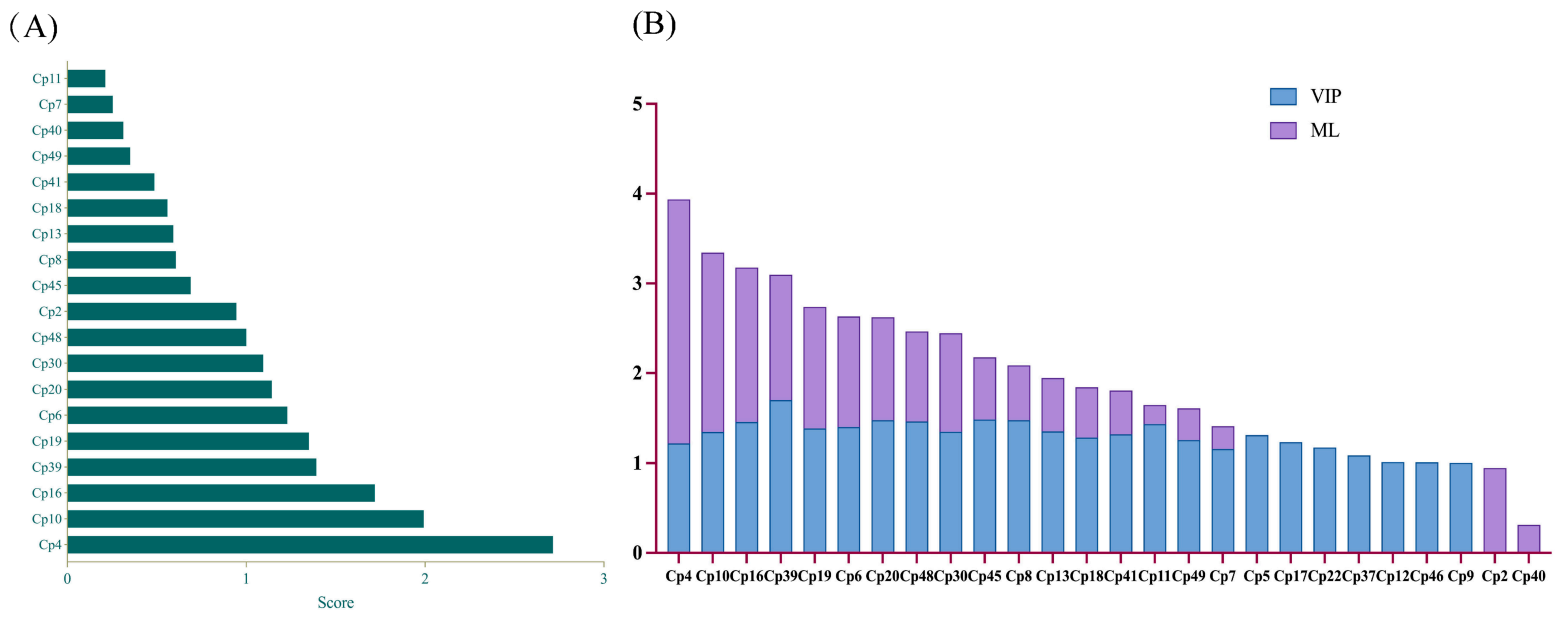
Integrated machine learning scores and composite VIP–SHAP scores of differential volatile compounds. Compound numbering is consistent with Table 1. (**A**) Integrated machine learning scores based on normalized SHAP values from the Random Forest, SVM, and CatBoost models; (**B**) composite scores obtained by combining normalized machine learning scores with PLS-DA VIP values.

To improve the robustness of candidate marker screening, the SHAP results from the Random Forest, SVM, and CatBoost models were integrated. The mean absolute SHAP value of each feature was first calculated and normalized within each model to reduce the influence of scale differences among models. The normalized values were then combined to obtain an integrated machine-learning contribution score (Figure 7A). This score was further combined with the VIP values from the PLS-DA model to generate a composite score, which considered both the contribution of each compound to PLS-DA-based group separation and its model-based contribution to sample differentiation (Figure 7B).

According to the composite score ranking, γ-hexalactone, methyl eugenol, methyl (9Z,11E)-octadeca-9,11-dienoate, eugenol, ethyl linoleate, 2-phenylethanol, benzyl alcohol, eugenol acetate, methyl linolenate, and 5-acetyl-2-methoxyphenyl acetate were identified as the top 10 candidate volatile markers associated with cultivation pattern-related differences in AR. Among them, γ-hexalactone showed the highest composite score (VIP = 1.2197, ML = 2.7157, composite score = 3.9354), indicating its strong contribution in both multivariate analysis and machine learning interpretation. These results suggest that the integrated VIP–SHAP strategy helped prioritize volatile compounds with stable and interpretable contributions to the quality-related volatile differences among wild, wild-simulated, and cultivated AR samples.

### 3.4. Candidate Discriminatory Volatile Compounds and Possible Metabolic Origin Associated with Cultivation-Pattern Differentiation of Astragali Radix

**Figure 8:**
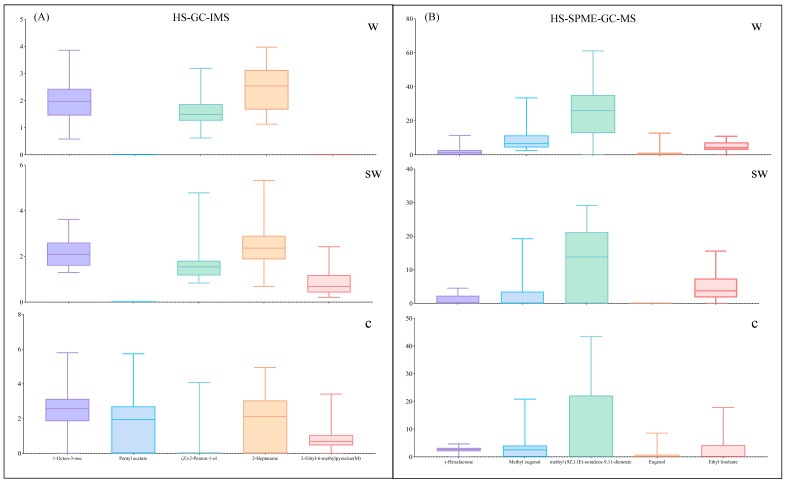
Boxplots of the relative contents of key differential volatile compounds in AR samples from different cultivation patterns. (**A**) Differential volatile compounds identified by HS-GC–IMS; (**B**) differential volatile compounds identified by HS-SPME-GC–MS. W, wild; SW, wild-simulated; C, cultivated.

By integrating VIP and SHAP analyses, γ-hexalactone, methyl eugenol, methyl (9Z,11E)-octadeca-9,11-dienoate, eugenol, and ethyl linoleate were selected as candidate discriminatory volatile compounds from the HS-SPME-GC–MS dataset, whereas 1-octen-3-one, pentyl acetate, (Z)-2-penten-1-ol, 2-heptanone, and the monomeric signal of 2-ethyl-6-methylpyrazine were selected from the HS-GC–IMS dataset. The relative abundances of these compounds showed different distribution patterns among wild, wild-simulated, and cultivated AR samples (Figure 8), suggesting that cultivation pattern was associated with variations in the volatile composition of AR. The HS-GC–IMS-derived candidate compounds mainly represented low-molecular-weight aldehydes, alcohols, and ketones and provided clearer group separation, whereas the HS-SPME-GC–MS-derived compounds covered more structurally complex medium- and high-boiling-point volatiles. However, the HS-SPME-GC–MS assignments remained tentative because authentic standards were not used, while HS-GC–IMS identification was constrained by the coverage of the retention-index and drift-time databases. These results demonstrate the complementary strengths and platform-specific limitations of the two analytical methods.

The candidate discriminatory compounds were mainly related to fatty acid-derived volatiles and aromatic secondary metabolites, with additional contributions from terpenoid-related compounds. Aldehydes, alcohols, ketones, esters, and lactones may arise from fatty acid oxidation, degradation, and downstream conversion. In particular, the lipoxygenase–hydroperoxide lyase pathway is involved in the formation of several aldehydes and alcohols associated with green-leaf volatiles [23]. Eugenol and methyl eugenol are phenylpropanoid/benzenoid volatiles associated with aromatic secondary metabolism, whereas terpenoid-related compounds, such as ar-turmerone and β-sesquiphellandrene, may originate from the mevalonate and methylerythritol phosphate pathways [24]. Differences in growth age, environmental exposure, cultivation management, and precursor availability may contribute to the observed volatile variation. However, these mechanistic interpretations remain hypothetical because targeted metabolomic or transcriptomic validation was not performed. In addition, because the wild and wild-simulated samples were collected only from Shanxi Province and were older than the cultivated samples, geographical origin and plant age were substantially confounded with cultivation pattern. Therefore, the observed volatile differences cannot be attributed solely to cultivation pattern [25]. Accordingly, the identified compounds should be regarded as candidate discriminatory features that may provide a basis for future cultivation-pattern authentication and volatile-profile-based differentiation of Astragali Radix.

## 4. Conclusions

Within the present sample set, cultivation pattern was associated with differences in the volatile fingerprints of Astragali Radix. The combined use of HS-SPME-GC–MS and HS-GC–IMS provided complementary information for characterizing volatile differences among wild, wild-simulated, and cultivated samples. HS-SPME-GC–MS was more suitable for detecting medium- and high-boiling-point volatiles with relatively complex structures, whereas HS-GC–IMS showed advantages in capturing low-molecular-weight and readily released compounds. Multivariate analysis and machine learning further supported cultivation-pattern-associated differentiation and helped prioritize candidate volatile contributors. By integrating VIP and SHAP analyses, γ-hexalactone, methyl eugenol, methyl (9Z,11E)-octadeca-9,11-dienoate, eugenol, and ethyl linoleate were selected as candidate discriminatory compounds from the HS-SPME-GC–MS dataset, whereas 1-octen-3-one, pentyl acetate, (Z)-2-penten-1-ol, 2-heptanone, and the monomeric signal of 2-ethyl-6-methylpyrazine were selected from the HS-GC–IMS dataset. These compounds may be related to fatty acid-derived, phenylpropanoid, and terpenoid-related metabolic processes. Overall, this study provides candidate discriminatory compounds and an interpretable volatile-fingerprinting strategy for differentiating Astragali Radix samples from different cultivation patterns. The integrated workflow may provide a laboratory-level analytical framework for preliminary cultivation-pattern authentication and candidate-compound screening, although further standardization and external validation are required before routine application. Several limitations should be acknowledged. The relatively small number of wild samples and the substantial confounding of geographical origin and plant age with cultivation pattern limit the causal attribution and generalizability of the present findings. Therefore, the observed differences should not be attributed solely to cultivation pattern. Future studies should include larger and geographically balanced sample sets and independently validate the candidate compounds and analytical workflow.

## Figures and Tables

**Table 1:** Differential volatile compounds with VIP values greater than 1 identified by HS-SPME-GC-MS.

Number	Compound	VIP	Normalized Peak Area-Based Relative Content (%)		
			w	sw	c
Cp39	Eugenol	1.703	0.016 ± 0.028	n.d.	0.0064 ± 0.013
Cp45	5-Acetyl-2-methoxyphenyl acetate	1.487	n.d.	n.d.	0.1275 ± 0.203
Cp8	Tetradecane	1.480	n.d.	0.0112 ± 0.012	0.01 ± 0.012
Cp20	Benzyl alcohol	1.479	0.0109 ± 0.018	0.0069 ± 0.013	0.033 ± 0.028
Cp48	Eugenol acetate	1.464	0.0093 ± 0.017	0.0025 ± 0.009	0.0034 ± 0.013
Cp16	methyl (9Z,11E)-octadeca-9,11-dienoate	1.458	0.2526 ± 0.15	0.1129 ± 0.112	0.1104 ± 0.14
Cp11	1,11-Hexadecadiyne	1.436	0.0053 ± 0.01	0.0501 ± 0.05	0.0383 ± 0.05
Cp6	2-Phenylethanol	1.402	0.0203 ± 0.029	0.0391 ± 0.042	0.0076 ± 0.009
Cp19	Ethyl linoleate	1.387	0.0465 ± 0.03	0.0486 ± 0.043	0.025 ± 0.041
Cp13	Methyl stearidonate	1.354	0.0099 ± 0.016	0.0276 ± 0.042	n.d.
Cp30	Methyl linolenate	1.350	0.1135 ± 0.097	0.0307 ± 0.052	0.033 ± 0.066
Cp10	Methyl eugenol	1.349	0.0922 ± 0.081	0.0226 ± 0.039	0.0258 ± 0.033
Cp41	Curlone	1.322	0.0314 ± 0.055	0.0318 ± 0.043	0.0102 ± 0.023
Cp5	Nonanal	1.313	n.d.	0.0192 ± 0.026	0.0135 ± 0.016
Cp18	Hexadecane	1.285	n.d.	0.0255 ± 0.035	0.0071 ± 0.012
Cp49	2-Phenoxyethanol	1.258	0.002 ± 0.004	0.0006 ± 0.002	0.0005 ± 0.002
Cp17	Naphthalene	1.235	0.0041 ± 0.009	0.0181 ± 0.033	0.0246 ± 0.03
Cp4	γ-Hexalactone	1.220	0.0185 ± 0.025	0.0112 ± 0.013	0.0258 ± 0.012
Cp22	Methyl hexanoate	1.175	n.d.	n.d.	0.0202 ± 0.027
Cp7	Nonanoic acid	1.158	0.0115 ± 0.013	0.0106 ± 0.017	0.0076 ± 0.009
Cp37	2-Methyl-3-octanol	1.087	n.d.	n.d.	0.0061 ± 0.01
Cp12	β-Sesquiphellandrene	1.013	0.0481 ± 0.06	0.156 ± 0.138	0.1331 ± 0.144
Cp46	Benzophenone	1.012	n.d.	n.d.	0.0015 ± 0.004
Cp9	2-Methoxy-4-vinylphenol	1.004	0.0236 ± 0.028	0.0277 ± 0.027	0.0144 ± 0.015

Abbreviations: w, wild; sw, wild-simulated; c, cultivated; n.d., not detected. Data are expressed as mean ± SD.

**Table 2:** Differential volatile compounds with VIP values greater than 1 identified by HS-GC-IMS.

Number	Compound	VIP	Normalized Peak Area-Based Relative Content (%)		
			w	sw	c
CP31/CP30	1-Octen-3-one	2.134/2.118	0.0197 ± 0.007	0.0219 ± 0.007	0.024 ± 0.014
CP146/CP145	Pentyl acetate	1.980/1.958	n.d.	n.d.	0.0185 ± 0.014
CP20/CP19	(Z)-2-Penten-1-ol	1.973/1.961	0.0164 ± 0.006	0.0172 ± 0.009	0.0045 ± 0.011
CP49/CP48	2-Heptanone	1.889/1.830	0.0245 ± 0.008	0.0249 ± 0.01	0.0174 ± 0.015
CP110	2-Ethyl-6-methylpyrazine(M)	1.813	n.d.	0.0084 ± 0.006	0.0091 ± 0.008
CP85/CP84	Butanal	1.363/1.345	0.0193 ± 0.007	0.0178 ± 0.006	n.d.
CP138/CP139	2-Butanol	1.359/1.344	n.d.	n.d.	0.0032 ± 0.009
CP136/CP137	Propyl butyrate	1.357/1.350	n.d.	n.d.	0.0047 ± 0.012
CP16/CP15	Nonanal	1.354/1.350	0.0207 ± 0.009	0.0224 ± 0.01	0.0188 ± 0.01
CP33/CP32	3-Octanone	1.351/1.350	0.022 ± 0.007	0.0184 ± 0.005	n.d.
CP7/CP8	2-Ethyl-1-hexanol	1.350/1.345	0.0149 ± 0.004	0.0149 ± 0.004	0.0042 ± 0.01
CP112/CP111	Eucalyptol	1.346/1.337	0.0146 ± 0.004	0.0212 ± 0.009	0.0238 ± 0.01
CP141/CP140	Tetrahydrofuran	1.341/1.340	n.d.	n.d.	0.0206 ± 0.01
CP124/CP123	Ethyl pyruvate	1.297/1.278	n.d.	0.0221 ± 0.008	0.0033 ± 0.011
CP4/CP3	Terpinen-4-ol	1.220/1.204	0.0248 ± 0.010	n.d.	n.d.
CP82/CP83	tert-Butanol	1.217/1.197	0.0198 ± 0.008	n.d.	n.d.
CP74/CP75	Isopropyl isobutyrate	1.217/1.214	0.0187 ± 0.004	n.d.	n.d.
CP80/CP81	Methacrolein	1.210/1.204	0.0202 ± 0.008	n.d.	n.d.
CP93/CP92	2,6-Dimethylpyrazine	1.209/1.196	0.0152 ± 0.007	0.0244 ± 0.011	n.d.
CP135/CP134	1,2-Dimethoxybenzene	1.206/1.190	n.d.	0.0187 ± 0.008	0.0038 ± 0.009
CP12/CP11	1-Octen-3-ol	1.205/1.197	0.0153 ± 0.005	n.d.	n.d.
CP72/CP73	2-Pentanone	1.205/1.196	0.0164 ± 0.006	n.d.	n.d.
CP115/CP116	(E, E)-2,4-Heptadienal	1.205/1.182	0.0175 ± 0.007	n.d.	0.0954 ± 0.023
CP131/CP132	2-Hexanol	1.204/1.204	n.d.	0.0211 ± 0.008	0.0153 ± 0.014
CP99/CP98	cis-3-Hexenyl acetate	1.204/1.197	0.0172 ± 0.004	n.d.	0.0214 ± 0.012
CP2/CP1	Butanoic acid	1.202/1.199	0.0202 ± 0.007	n.d.	n.d.
CP88/CP89	1-Octene	1.199/1.191	0.0189 ± 0.01	n.d.	n.d.
CP25/CP24	2-Octanone	1.198/1.195	0.0163 ± 0.005	n.d.	n.d.
CP87/CP86	Methyl propionate	1.196/1.188	0.0138 ± 0.006	n.d.	n.d.
CP91/CP90	Propanal	1.195/1.186	0.0152 ± 0.008	n.d.	n.d.
CP9/CP10	Methyl 2-furoate	1.161/1.144	0.0137 ± 0.005	n.d.	0.004 ± 0.014
CP45/CP44	2-Methyl-1-butanol	1.039/1.020	0.0188 ± 0.007	n.d.	0.0321 ± 0.01
CP117/CP118	Propylene glycol	1.031/1.013	n.d.	0.0184 ± 0.006	n.d.
CP35/CP34	Butyl isovalerate	1.030/1.005	0.0201 ± 0.007	n.d.	0.0165 ± 0.013
CP38/CP39	Ethyl hexanoate	1.028/1.001	0.017 ± 0.007	n.d.	0.0231 ± 0.01
CP119/CP120	2-Methylbutanoic acid	1.026/1.023	n.d.	0.0271 ± 0.011	n.d.
CP129/CP130	2,2,4,6,6-Pentamethylheptane	1.026/1.015	n.d.	0.0234 ± 0.009	n.d.
CP128/CP127	3-Hexanone	1.024/1.012	n.d.	0.0191 ± 0.008	n.d.
CP121/CP122	Benzaldehyde	1.024/1.022	n.d.	0.0222 ± 0.008	n.d.
CP126/CP125	Butyl acrylate	1.020/1.012	n.d.	0.0175 ± 0.007	n.d.

Abbreviations: w, wild; sw, wild-simulated; c, cultivated; n.d., not detected. Data are expressed as mean ± SD.

## Data Availability

The original contributions presented in this study are included in the article/Supplementary Materials. Further inquiries can be directed to the corresponding author.

## Supplementary Materials

The following supporting information can be downloaded at: https://www.mdpi.com/article/10.3390/foods15152624/s1, Table S1:Volatile compounds identified by HS-SPME-GC-MS in Astragali Radix samples with different growth patterns; Table S2: Volatile compounds identified by HS-GC-IMS in Astragali Radix samples with different growth patterns; Table S3: Analytical repeatability of representative HS-GC–IMS peaks based on triplicate measurements; Table S4: Analytical repeatability of representative HS-SPME-GC–MS peaks based on triplicate measurements.
