# Supplementary material for: Volatile Fingerprinting and Interpretable Machine Learning for Quality Differentiation of Astragali Radix from Different Cultivation Patterns
Source: Foods. 2026 Jul 27;15(15):2624. doi: 10.3390/foods15152624 (PMC13465424; doi:10.3390/foods15152624)
Supplement: Supplementary file 1 [file foods-15-02624-s001.zip › foods-4426261-supplementary.pdf]

Table S1. Volatile compounds identified by HS-SPME-GC-MS in Astragali Radix samples with different growth patterns

Compound	Cas	Normalized peak area–based relative abundance (%)		
		w	sw	c
2-Methyl-3-octanol	26533-34-6	n.d.	n.d.	0.006±0.010
1-Nonen-4-ol	35192-73-5	n.d.	n.d.	0.0030±0.0080
δ-Cadinol	19435-97-3	n.d.	n.d.	0.011±0.036
2-Phenoxyethanol	122-99-6	0.0020±0.0040	0.0006±0.0020	0.0005±0.0020
1-Hexanol	111-27-3	0.029±0.026	0.034±0.029	0.020±0.022
2-Phenylethanol	60-12-8	0.020±0.029	0.039±0.042	0.0076±0.0090
Benzyl alcohol	100-51-6	0.011±0.018	0.007±0.013	0.033±0.028
1-Hexadecanol	36653-82-4	0.011±0.016	0.014±0.013	0.017±0.021
1-Heptyn-3-ol	7383-19-9	n.d.	0.0045±0.0090	0.0009±0.0030
2-Ethyl-1-hexanol	104-76-7	n.d.	n.d.	0.006±0.012
2-Methoxy-4-vinylphenol	7786-61-0	0.024±0.028	0.028±0.027	0.014±0.015
Paeonol	552-41-0	n.d.	n.d.	0.0011±0.0040
Eugenol	97-53-0	0.016±0.028	n.d.	0.006±0.013
Methyl eugenol	93-15-2	0.092±0.081	0.023±0.039	0.026±0.033
Isoelemicin	487-12-7	n.d.	n.d.	0.0011±0.0030
Hexanal	66-25-1	0.029±0.021	0.077±0.048	0.052±0.022
Nonanal	124-19-6	n.d.	0.019±0.026	0.014±0.016
5-Formyl-2-methoxyphenyl acetate	881-57-2	0.004±0.010	0.006±0.013	0.0017±0.0070
(E, E)-2,4-Nonadienal	5910-87-2	n.d.	0.0023±0.0060	n.d.
Nonanoic acid	112-05-0	0.012±0.013	0.011±0.017	0.0076±0.0090
Palmitic acid	57-10-3	0.013±0.025	0.006±0.014	0.006±0.020
Pentanoic acid	109-52-4	n.d.	n.d.	0.0018±0.0040
8-Methylnonanoic acid	5963-14-4	n.d.	n.d.	0.0019±0.0050
Tetradecane	629-59-4	n.d.	0.011±0.012	0.010±0.012
1,11-Hexadecadiyne	71673-32-0	0.005±0.010	0.050±0.050	0.038±0.050
Naphthalene	91-20-3	0.0041±0.0090	0.018±0.033	0.025±0.030
Hexadecane	544-76-3	n.d.	0.026±0.035	0.007±0.012
β-Sesquiphellandrene	20307-83-9	0.048±0.060	0.16±0.14	0.13±0.14
2,6,10-Trimethyltridecane	3891-99-4	n.d.	0.007±0.017	n.d.
(E, E)-3,5-Octadien-2-one	38284-27-4	0.028±0.030	0.062±0.045	0.040±0.032
4-Acetyl-1-methylcyclohexene	6090-09-1	n.d.	n.d.	0.0020±0.0050
Curlone	87440-60-6	0.031±0.055	0.032±0.043	0.010±0.023
ar-Turmerone	532-65-0	0.08±0.12	0.10±0.13	0.042±0.050
Benzophenone	119-61-9	n.d.	n.d.	0.0015±0.0040
Anthraquinone	84-65-1	n.d.	n.d.	0.0025±0.0090
5-Acetyl-2-methoxyphenyl acetate	60792-88-3	n.d.	n.d.	0.13±0.20

Compound	Cas	Normalized peak area–based relative abundance (%)		
		w	sw	c
Methyl formate	107-31-3	0.016±0.028	0.007±0.014	0.045±0.068
Methyl hexanoate	106-70-7	0.0035±0.0040	0.008±0.010	0.0025±0.0040
methyl (9Z,11E)-octadeca-9,11-dienoate	13058-52-1	0.25±0.15	0.11±0.11	0.11±0.14
Ethyl linoleate	544-35-4	0.047±0.030	0.049±0.043	0.025±0.041
Ethyl palmitate	628-97-7	0.007±0.010	0.008±0.014	0.0034±0.0090
Methyl palmitate	112-39-0	n.d.	n.d.	0.020±0.027
Methyl linolenate	301-00-8	0.114±0.097	0.031±0.052	0.033±0.066
Methyl salicylate	119-36-8	n.d.	n.d.	0.0010±0.0030
Eugenol acetate	93-28-7	0.009±0.017	0.0025±0.0090	0.003±0.013
Methyl stearidonate	73097-00-4	0.010±0.016	0.028±0.042	n.d.
Methyl palmitelaidate	10030-74-7	0.0012±0.0030	n.d.	n.d.
Methyl palmitoleate	1120-25-8	n.d.	0.004±0.010	n.d.
γ-Hexalactone	695-06-7	0.019±0.025	0.011±0.013	0.026±0.012
γ-Nonalactone	104-61-0	0.060±0.077	n.d.	0.047±0.099
Dehydrocostus lactone	477-43-0	n.d.	0.0042±0.0090	n.d.
2,4-Dihydroxy-2,5-dimethyl-3(2H)-furanone	10230-62-3	0.005±0.015	0.0013±0.0050	0.008±0.059
1-(2-methyloxolan-2-yl) ethan-1-one	32318-87-9	0.0012±0.0060	n.d.	n.d.

Abbreviations: w, wild; sw, semi-wild; c, cultivated; n.d., not detected. Data are expressed as mean ± SD.

Table S2. Volatile compounds identified by HS-GC-IMS in Astragali Radix samples with different growth patterns

Compound	Cas	RI	Normalized peak area–based relative content (%)		
			w	sw	c
Terpinen-4-ol	562-74-3	1620.1-1621.6	0.025±0.010	n.d.	n.d.
2-Ethyl-1-hexanol	104-76-7	1499.6-1509.8	0.0149±0.0040	0.0149±0.0040	0.004±0.010
1-Octen-3-ol	3391-86-4	1459.8-1462.2	0.0153±0.0050	n.d.	n.d.
1-Hexanol	111-27-3	1357.6-1364.1	0.0129±0.0030	0.0187±0.0060	0.005±0.010
(Z)-2-Penten-1-ol	1576-95-0	1328.1-1332.9	0.0164±0.0060	0.0172±0.0090	0.005±0.011
1-Pentanol	71-41-0	1257.9-1308.7	0.0119±0.0020	0.0159±0.0040	0.0184±0.0090
2-Methyl-1-butanol	137-32-6	1204.3-1208.3	0.0188±0.0070	n.d.	0.032±0.010
3-Methyl-1-butanol	123-51-3	1205.8-1264.0	0.0193±0.0060	0.0213±0.0080	0.030±0.011
1-Penten-3-ol	616-25-1	1153.3-1158.1	0.0139±0.0070	0.0163±0.0070	0.022±0.012
2-Pentanol	6032-29-7	1153.5-1158.1	0.0196±0.0050	0.0225±0.0080	0.010±0.012
1-Butanol	71-36-3	1138.0-1141.8	0.0225±0.0090	0.0271±0.0080	0.028±0.013
2-Methyl-1-propanol	78-83-1	1090.8-1094.0	0.0198±0.0070	0.025±0.010	0.024±0.013
1-Propanol	71-23-8	1032.2-1043.7	0.0115±0.0050	0.024±0.012	0.018±0.013
tert-Butanol	75-65-0	894.4-898.5	0.0198±0.0080	n.d.	n.d.

Compound	Cas	RI	Normalized peak area–based relative content (%)		
			w	sw	c
Propylene glycol	57-55-6	1635.4-1636.3	n.d.	0.0184±0.0060	n.d.
2-Hexanol	626-93-7	1262.2-1304.6	n.d.	0.0211±0.0080	0.015±0.014
2-Butanol	78-92-2	1023.5-1036.8	n.d.	n.d.	0.0032±0.0090
trans-2-Hexen-1-ol	2305-21-7	1374.8-1375.1	n.d.	n.d.	0.005±0.017
3-Methyl-1-pentanol	589-35-5	1343.9-1344.1	n.d.	n.d.	0.005±0.015
Ethanol	64-17-5	953.1-962.4	0.0177±0.0060	0.0203±0.0050	0.028±0.014
2,6-Dimethoxyphenol	91-10-1	1342.2-1346.2	n.d.	0.0187±0.0080	0.0038±0.0090
Eucalyptol	470-82-6	1197.1-1264.6	0.0146±0.0040	0.0212±0.0090	0.024±0.010
Rose oxide	16409-43-1	1358.7-1398.4	0.0152±0.0070	0.024±0.011	n.d.
2-Pentylfuran	3777-69-3	1232.5-1235.7	n.d.	0.0084±0.0060	0.0091±0.0080
(E)-2-Octenal	2548-87-0	1418.8-1430.8	0.0176±0.0090	0.0194±0.0080	0.026±0.011
Nonanal	124-19-6	1389.1-1395.7	0.0207±0.0090	0.022±0.010	0.019±0.010
(E)-2-heptenal	18829-55-5	1324.5-1331.0	0.023±0.010	0.024±0.011	0.025±0.013
3-Methyl-2-butenal	107-86-8	1199.4-1204.3	0.0170±0.0060	0.0246±0.0070	0.022±0.012
2-Hexenal	505-57-7	1201.7-1225.5	0.0146±0.0060	0.0198±0.0080	0.0183±0.0090
Heptanal	111-71-7	1179.2-1184.6	0.0185±0.0070	0.0241±0.0090	0.022±0.010
Hexanal	66-25-1	1078.3-1134.2	0.0181±0.0060	0.0215±0.0040	0.023±0.012
Pentanal	110-62-3	979.4-991.9	0.0196±0.0070	0.0182±0.0040	0.024±0.012
Methacrolein	78-85-3	887.5-887.8	0.0202±0.0080	n.d.	n.d.
Octanal	124-13-0	1292.6-1296.4	0.0174±0.0090	0.0155±0.0060	0.024±0.015
trans-2-Hexenal	6728-26-3	1219.8-1225.1	0.0176±0.0070	0.0168±0.0050	0.0147±0.0070
trans-2-Pentenal	1576-87-0	1128.6-1132.8	0.0179±0.0080	0.0186±0.0060	0.017±0.011
2-Methyl-2-pentenal	623-36-9	1146.0-1149.9	0.0142±0.0050	0.023±0.010	0.033±0.015
(E,E)-2,4-Heptadienal	4313-03-5	1482.0-1488.1	0.0175±0.0070	n.d.	0.030±0.015
Benzaldehyde	100-52-7	1559.1-1559.6	n.d.	0.0222±0.0080	n.d.
Butanal	123-72-8	882.3-889.7	0.0193±0.0070	0.0178±0.0060	n.d.
Propanal	123-38-6	777.7-778.9	0.0152±0.0080	n.d.	n.d.
2,4-Heptadienal	5910-85-0	1521.3-1521.6	n.d.	n.d.	0.003±0.011
Butanoic acid	107-92-6	1689.6-1690.0	0.0202±0.0070	n.d.	n.d.
Propanoic acid	79-09-4	1555.4-1575.2	0.0142±0.0060	0.0175±0.0040	0.0232±0.0080
2-Methylpropanoic acid	79-31-2	1618.7-1634.0	0.0197±0.0090	0.0173±0.0080	0.021±0.011
2-Methylbutanoic acid	116-53-0	1621.5-1622.1	n.d.	0.027±0.011	n.d.
Ethylbenzene	100-41-4	1173.2-1186.2	0.0182±0.0080	0.0177±0.0080	0.028±0.013
1-Octene	111-66-0	873.8-881.3	0.019±0.010	n.d.	n.d.
2,2,4,6,6-Pentamethylheptane	13475-82-6	921.4-922.4	n.d.	0.0234±0.0090	n.d.
1-Hydroxy-2-propanone	116-09-6	1269.1-1312.1	0.0185±0.0070	0.023±0.010	0.027±0.013
6-Methyl-5-hepten-2-one	110-93-0	1323.2-1340.0	0.0084±0.0040	0.0104±0.0070	0.005±0.016
2-Octanone	111-13-7	1329.8-1330.6	0.0163±0.0050	n.d.	n.d.
Acetoin	513-86-0	1289.9-1295.5	0.0161±0.0070	0.0191±0.0050	0.019±0.010

Compound	Cas	RI	Normalized peak area–based relative content (%)		
			w	sw	c
Cyclohexanone	108-94-1	1307.8-1310.0	0.0174±0.0080	0.0193±0.0070	0.021±0.012
1-Octen-3-one	4312-99-6	1304.5-1344.7	0.0197±0.0070	0.0219±0.0070	0.024±0.014
3-Octanone	106-68-3	1301.9-1303.3	0.0220±0.0070	0.0184±0.0050	n.d.
2-Heptanone	110-43-0	1174.9-1185.0	0.0245±0.0080	0.025±0.010	0.017±0.015
4-Heptanone	123-19-3	1153.5-1158.7	0.0168±0.0050	0.019±0.010	0.025±0.013
1-Penten-3-one	1629-58-9	994.8-1026.5	0.0139±0.0040	0.0179±0.0050	0.029±0.018
2-Pentanone	107-87-9	977.7-983.4	0.0164±0.0060	n.d.	n.d.
3-Penten-2-one	625-33-2	1127.0-1128.4	n.d.	0.0191±0.0080	n.d.
2-Decanone	693-54-9	1475.2-1477.4	n.d.	n.d.	0.005±0.015
4-Methylcyclohexanone	589-92-4	1344.0-1344.4	n.d.	n.d.	0.007±0.021
Cyclopentanone	120-92-3	1186.5-1189.9	n.d.	n.d.	0.004±0.012
Methyl 2-furoate	611-13-2	1510.2-1533.3	0.0137±0.0050	n.d.	0.004±0.014
Ethyl pyruvate	617-35-6	1250.1-1266.5	n.d.	0.0221±0.0080	0.003±0.011
Butyl isovalerate	109-19-3	1280.6-1283.1	0.0201±0.0070	n.d.	0.017±0.013
Ethyl hexanoate	123-66-0	1237.5-1242.5	0.0170±0.0070	n.d.	0.023±0.010
Isopropyl isobutyrate	617-50-5	974.1-990.3	0.0187±0.0040	n.d.	n.d.
Methyl propionate	554-12-1	870.4-872.7	0.0138±0.0060	n.d.	n.d.
Methyl hexanoate	106-70-7	1180.5-1188.2	0.020±0.010	0.021±0.011	0.022±0.012
Butyl acrylate	141-32-2	1171.3-1171.4	n.d.	0.0175±0.0070	n.d.
Propyl butyrate	105-66-8	1169.0-1173.9	n.d.	n.d.	0.005±0.012
cis-3-Hexenyl acetate	3681-71-8	1314.5-1318.3	0.0172±0.0040	n.d.	0.021±0.012
Pentyl acetate	628-63-7	1169.6-1170.7	n.d.	n.d.	0.019±0.014
γ-Butyrolactone	96-48-0	1614.8-1639.2	0.021±0.010	0.0204±0.0060	0.023±0.012
Tetrahydrofuran	109-99-9	877.3-888.2	n.d.	n.d.	0.021±0.010

Abbreviations: w, wild; sw, semi-wild; c, cultivated; n.d., not detected. Data are expressed as mean ± SD.

Table S3. Analytical repeatability of representative HS-GC–IMS peaks based on triplicate measurements.

**Compound**	**Median RSD (%)**
1-Penten-3-ol	6.23
1-Hexanol	6.00
1-Pentanol	7.27
4-Heptanone	6.32
Heptanal	8.57
1-Propanol	5.74
Pentanal	5.18
Ethanol	6.60

Methyl propionate	8.19
(E)-2-Octenal	7.07

Representative peaks were selected based on a detection frequency greater than 95%. For each sample, the RSD was calculated from three independent analytical replicates, and the median RSD across the detected samples is reported.

Table S4. Analytical repeatability of representative HS-SPME-GC–MS peaks based on triplicate measurements.

**Compound**	**Median RSD (%)**
Hexanal	8.56
1-Hexanol	5.51
γ-Hexalactone	7.83
(E, E)-3,5-Octadien-2-one	5.78
2-Methoxy-4-vinylphenol	4.46
Methyl eugenol	6.34
β-Sesquiphellandrene	4.75
Benzyl alcohol	7.92

Representative peaks were selected based on a detection frequency greater than 70%. For each sample, the RSD was calculated from three independent analytical replicates, and the median RSD across samples in which the peak was detected in all three replicates is reported.
